# Predictors of early initiation of breastfeeding in women who give birth in Sri Lankan hospitals: A cross-sectional assessment of socio-demographic and clinical measures

**DOI:** 10.1371/journal.pone.0326863

**Published:** 2025-07-02

**Authors:** Laavanya Lokeesan, Elizabeth Martin, Yvette D. Miller

**Affiliations:** 1 Centre for Health Care Transformation, School of Public Health and Social Work, Faculty of Health, Queensland University of Technology, Brisbane, Queensland, Australia; 2 Wesley Research Institute, Brisbane, Queensland, Australia; 3 Mater Research Institute, University of Queensland, Brisbane, Queensland, Australia; Flinders University, AUSTRALIA

## Abstract

**Background:**

Early initiation of breastfeeding within one hour of birth can be predicted by socio-demographic and clinical characteristics of women.The relative influence of many socio-demographic and clinical characteristics on early initiation of breastfeeding has not been established in Sri Lanka. Identifying the significant predictors of early initiation of breastfeeding in Sri Lankan women may influence the Sri Lankan health system to review and renew policies and practices to support women who are at risk of delayed initiation of breastfeeding.

**Methods:**

A cross-sectional survey was conducted with women who had a live baby across selected hospitals in Sri Lanka. Women who were 12 hours post-birth and still admitted to the hospital were invited to participate. Data were collected by interviewing participants and extracting additional clinical information from their medical records. The contribution of socio-demographic and clinical characteristics to explaining variations in early initiation of breastfeeding was estimated using binary logistic regression analysis with simultaneous adjustment.

**Results:**

The rate of early initiation of breastfeeding was 64.5% (n = 195). Participants with a body mass index under 18.5 kg/m^2^ (OR: 4.17; 95% CI: 1.23–14.05) were more likely to practice early initiation of breastfeeding, and less likely if they gave birth by elective caesarean section (OR: 0.27; 95% CI: 0.14–0.51), were administered antibiotics (OR: 0.4; 95% CI: 1.05–4.73) or had a baby with respiratory distress (OR: 0.08, 95% CI: 0.01–0.57).

**Conclusion:**

Current intrapartum care practices associated with elective caesarean and antibiotic administration, and immediate care of babies with respiratory distress, should be critically reviewed to understand the mechanisms underlying their negative impact on breastfeeding initiation in Sri Lanka. Compliance with breastfeeding support care should be monitored to provide equal care for women,  minimising discrepancies in early initiation of breastfeeding associated with clinical circumstances.

## Background

Initiation of breastfeeding is the foundation of successful breastfeeding and is critical to infant health [1]. The World Health Organization [WHO] and United Nations International Children’s Emergency Fund (UNICEF) [2] recommend initiation of breastfeeding within one hour of birth, known as early initiation of breastfeeding, to ensure infants receive colostrum. Colostrum is the “first milk” secreted by the human breast [3,4] containing protective and growth-promoting factors [5] that improve immunity and development in babies [3,5] Initiation of breastfeeding within one hour of birth reduces the risk of death in babies during the first month of their life [4] and increases the likelihood of exclusive breastfeeding to six months of age.

Inconsistencies in early initiation of breastfeeding have been reported across the world and are associated with the socio-demographic profile of women, pregnancy and childbirth characteristics, and healthcare support for breastfeeding [3,4]. Globally, women living in urban areas, aged 20–35 years, with a higher level of education, unemployed, married, primiparous, without complications during pregnancy and after birth, who are not administered medications during labor, and who have babies after 37 weeks gestation without complications are more likely to initiate breastfeeding early [3,6–8]. However, associations between early initiation of breastfeeding and many socio-demographic and clinical characteristics have not been established in Sri Lanka. What has been established is that early breastfeeding is more likely with babies who weighed over 2500 grams at birth, who were born vaginally, in government hospitals, and who were home-visited by a Public Health Midwife [PHM] [9], consistent with global literature [3,4]. The role of a wider range of possible circumstances that explain variations in early initiation of breastfeeding is not clear in Sri Lanka [10].

Sri Lankan women are from multi-ethnic and socio-demographic backgrounds and receive health services from different types of health facilities [11]. Understanding the determinants of early initiation of breastfeeding in Sri Lankan women may help to identify the characteristics of women who are least likely to initiate breastfeeding early. These women and circumstances can therefore be targeted with interventions to support early initiation of breastfeeding across Sri Lanka. In this research, we aimed to identify significant predictors of early initiation of breastfeeding from a wide range of women’s socio-demographic and clinical characteristics related to pregnancy and childbirth. We also sought to assess the relative contribution of these variables to explaining variations in early initiation of breastfeeding so that priority sub-populations and points of intervention for early breastfeeding support could be identified.

## Materials and methods

### Study design and setting

**Fig 1:**
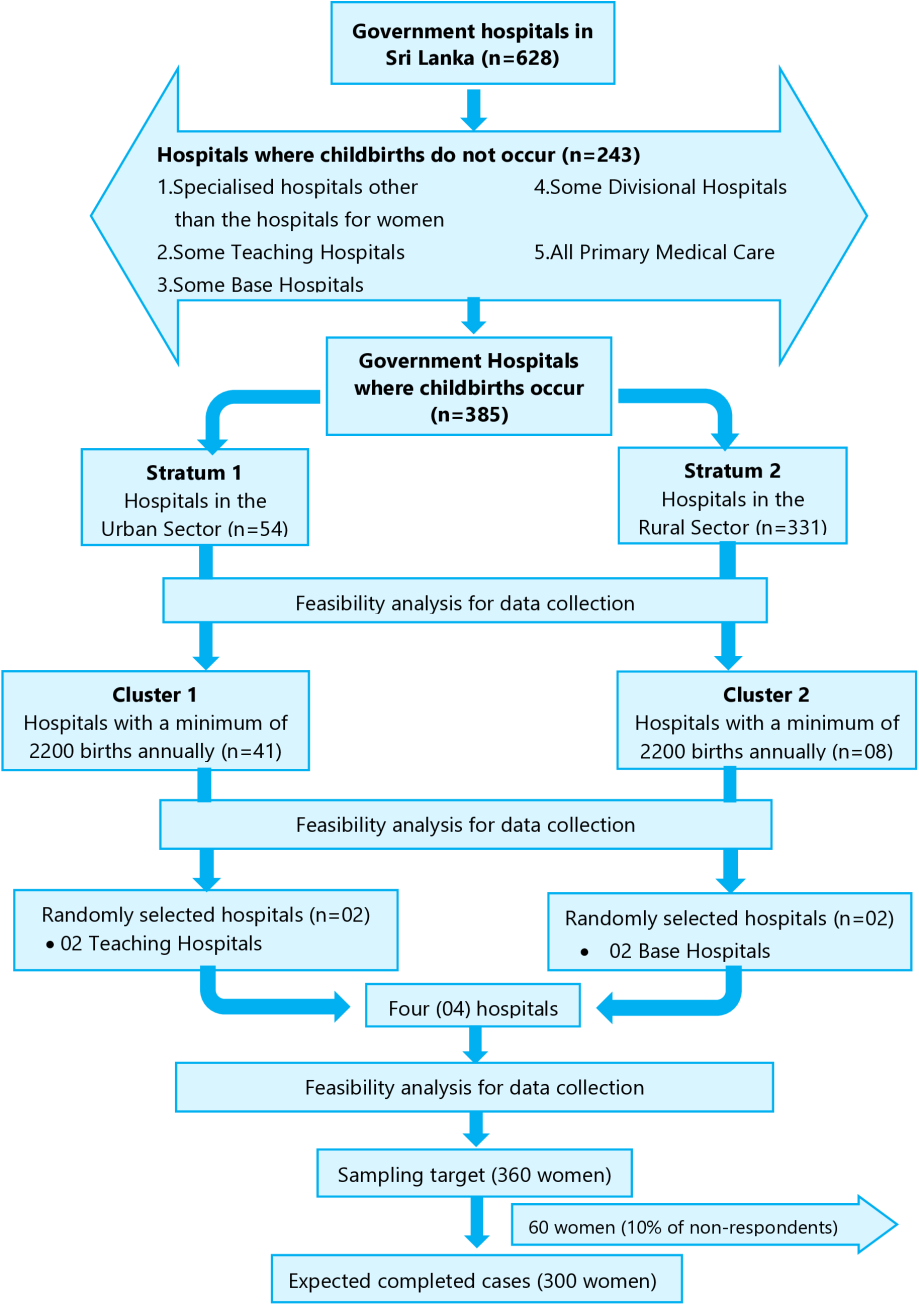
Sampling Framework.

A cross-sectional survey was conducted from 20^th^ September to 21^st^ December 2021 in four Sri Lankan government hospitals. In Sri Lanka, 94% of births occur in government hospitals, and they are the major maternity care service providers [12,13]. Stratified cluster random sampling was applied to select hospitals from which to draw the study sample (Fig 1). Government hospitals in Sri Lanka where women give birth (n = 385) were stratified into two groups to represent both larger and smaller hospitals in our study. Stratum 1 was hospitals in the urban sector of Sri Lanka (n = 54) which are larger and stratum 2 was hospitals in the rural sector (*n* = 331) which are smaller than the hospitals in the urban sector. Considering feasibility discussed below in the section ‘sample size’, to approach a minimum of six postpartum women in any day during the data collection period, a minimum of six births per day in that hospital [or at least 2200 births in a year (> 365 × 6 = 2190)] were necessary. Therefore, based on the available annual birth data, hospitals with a minimum of 2200 births in 2018 (n = 49) [13] were clustered from each stratum to increase sampling efficiency. This meant we could collect the largest amount of data from women admitted to each hospital during the limited data collection period. Clusters of strata 1 and 2 included 41 and 8 hospitals, respectively. Two hospitals were randomly selected from each cluster.

### Participants

Women who (1) had live single or multiple births from 19^th^ September to 20^th^ December 2021 in selected hospitals, (2) did not have a neonatal death as women with neonatal deaths might not have initiated breastfeeding, (3) could understand and speak one of three languages (Sinhala/Tamil/English), and (4) were physically and mentally capable of consenting to participate in this study, were approached and invited to participate at least 12 hours after birth. Women were eligible to participate until discharged from the hospital.

### Sample size

The required sample size for regression analysis was determined according to Green’s rule of thumb [14] that N > 50 + 8m is needed for multiple correlations and N > 104 + m is needed for testing individual predictors, where ‘N’ is the required sample size, and ‘m’ is the number of independent variables (18 in our study). According to Green’s [14] rule, the minimum number needed for testing multiple correlations and individual predictors was 195 [N > 50+(8 × 18) =N > 194] and 123 [N > 104 + 18 = N > 122] respectively. Data collection feasibility was also considered. The principal researcher had 60 days available for data collection, approximately four hours permitted by the hospitals for data collection each day, and 30–40 minutes planned with each participant for face-to-face interviews and clinical data extraction. Approximately six women could be approached each day, with five women expected to participate based on a 90% estimated response [15]. Therefore, the research team aimed to approach 360 women to achieve an expected sample of 300 (Fig 1), providing sufficient power and adequacy for planned statistical analyses after accounting for potential non-consent missing or invalid data. The samples were divided among the four selected hospitals proportionally according to the number of births that occurred in the selected hospitals in 2018 [13].

### Ethics approval

Ethics approval to conduct this study was obtained from the University Human Research Ethics Committee of the Queensland University of Technology, Australia (Approval number – 2000001001), Ethics Review Committee of Faculty of Health-Care Sciences, Eastern University, Sri Lanka (Application number – E/2021/05), and the Ethics Review Committee of Department of Health Services, North-Western province, Sri Lanka (Application number – ERC/NWP/2021/11).

### Data collection

A structured electronic survey instrument on Key Survey [16], including a questionnaire and data entry tool, was used to collect self-reported data from post-partum women and clinical data from their medical records. Survey questions asked women’s socio-demographic details, and information about their pregnancy, labor and childbirth, and infant feeding practices.

A healthcare worker who was either the nurse in charge or one of the nursing officers of the postnatal wards of the selected hospitals assisted the principal researcher in identifying eligible women by providing their bed numbers. The health care worker also informed eligible women about the researcher’s presence in the postnatal ward, and that the researcher was independent from hospital staff*.* The principal researcher approached eligible women, briefly explained the purpose of this research and the data collection procedure and provided the participant with an information sheet to read and understand more about the study. The principal researcher is competent in speaking both Tamil and Sinhala languages and, therefore, according to women’s preference, the researcher conducted face-to-face interviews either in Tamil or Sinhala with those who provided written consent to participate (n = 302) in this study. An interview with one participant lasted approximately 20–30 minutes. The researcher interviewed each woman at their bedside and recorded participants’ responses to the survey. The principal researcher obtained information from women about colostrum feeding and their socio-demographic characteristics using the survey questionnaire. Also, the researcher obtained data on clinical measures for participants’ most recent pregnancy, birth, and obstetric history from participants’ medical records, and recorded them in the survey.

Aggregate data for all women who had live babies from September to December 2021 were accessed from birth registers maintained in labor rooms of the selected hospitals to assess sample representativeness. National data for women who gave birth in Sri Lanka in the previous years were obtained from national reports that were openly available on the official websites of the Family Health Bureau, Sri Lanka, and the Department of Census and Statistics, Sri Lanka, to compare participants’ characteristics with the national population women who give birth.

### Measures

#### Early initiation of breastfeeding.

Early initiation of breastfeeding is the outcome variable of this study, which was assessed as breastfeeding within one hour of birth. Participants were asked when they started feeding colostrum for the very first time after giving birth. Responses were recorded as less than 10 minutes after birth, 10 to 30 minutes after birth, and 31 to 60 minutes after birth, later combined into “early initiation of breastfeeding”. More than 1 hour and not yet initiated were re-coded as “no early initiation of breastfeeding” [17]. All responses of eleven women who responded “do not know or do not remember” were excluded from analyses as women’s responses could not be meaningfully  categorised for the outcome, and they introduce bias in measuring early initiation of breastfeeding.

#### Socio-demographic characteristics.

Independent variables were women’s socio-demographic and clinical characteristics. Socio-demographic factors collected via maternal self-report using the survey questionnaire included age, area of residence, level of education, employment, annual household income, ethnicity, and relationship status. Maternal age and household income were collected continuously and later categorized into five categories for age (“15-19 years”, “20-24 years”, “25-29 years”, “30-34 years”, and “35 years and older”) and four categories for income (“<100, 000”, “100, 000–500,000”, “501,000–1,000,000”, and “> 1,000,000” Sri Lankan Rupees). Self-reported data obtained from women regarding antenatal clinic visits were transformed into dichotomous variables (“visited” and “not visited”).

#### Clinical measures.

Data on clinical characteristics included pregnancy (parity, pregnancy complications) and childbirth (mode of birth, gestational age, baby’s birth weight, postpartum maternal complications, neonatal complications) were obtained from participants’ medical records. Dichotomous variables sourced from clinical data were derived for parity (“primipara” and “multiparous”), infant gestational age at birth (“<37 weeks” and “≥37 weeks”), and infant birthweight (“< 2500 grams” and “≥2500 grams”), consistent with standards commonly used to assess associations with early initiation of breastfeeding. Standard Body Mass Index (BMI) categories for Asians were used to define pre-pregnancy body mass index [BMI] as “<18.5 kg/m^2^, “18.5-22.9 kg/m^2^”, “23-27.49 kg/m^2^”, and “≥27.5 kg/m^2^” [9]. Dichotomous variables of “administered” and “not administered” were derived for assessing the administration of medications, and “presented” and “not presented” were derived for pregnancy, birth, and postpartum complications. Mode of birth was categorized into “unassisted vaginal birth”, “vaginal birth assisted with forceps”, vaginal birth assisted with the vacuum”, “vaginal birth assisted with both forceps and vacuum”, “elective caesarean section” and “emergency caesarean section”.

### Statistical analysis

Data analysis was conducted with Statistical Package for Social Sciences (SPSS) for Windows version 28.0.1. Sample representativeness was determined by comparing participants’ characteristics with different populations for which comparable data was available: a) women who gave birth to a live baby in the selected hospitals from September to December 2021, b) live births that were registered in Sri Lanka in 2015 [18], and c) live births reported in 2019 [11]. This approach enabled the assessment of the generalis ability of the study sample to the national population and women who give birth in the selected hospitals. Characteristics of participants were compared with populations using one-sample chi-square tests.

Multivariable logistic regression was used to identify independent significant predictors of early initiation of breastfeeding. Socio-demographic characteristics and clinical measures related to pregnancy and childbirth were added to the model as independent variables. The association between early initiation of breastfeeding and every independent variable was assessed with univariate logistic regression, followed by multivariable modelling for all variables simultaneously using binary logistic regression with step-wise backward elimination to estimate odds ratios (OR) after adjustment for independent variables. Residuals and standard errors for multicollinearity were examined for independent variables. Variables that were significant at α < 0.05 were retained in the final model and results were expressed as crude and adjusted odd ratios [aOR] of early initiation of breastfeeding, with 95% confidence intervals (CI).

## Results

Of the 360 eligible women who were invited, 302 (83.8%) consented to participate. There were no missing data in this sample. Participants’ characteristics are presented in Table 1. The majority of participants were aged 30–34 years (n = 89, 29.5%), were Sinhalese (n = 199, 65.9%), had secondary education (n = 258, 85.4%), were unemployed (n = 240, 79.5%), had previously given birth to a live baby (n = 173, 57.3%), and had given birth to a single, term baby this time (n = 282, 93.4%).

**Table 1:** Participants’ characteristics and their distribution compared to relevant populations.

Characteristics	Number	Percentage (n = 302)	Percentage in total population			*p-value*
			^a^(n = 4497)	^b^(n = 336,097)	(n = 294,504)^c†^ (n = 301,265)^c††^ (n = 341,745)^c†††^	
***Socio-demographics***						
Age						<0.001^*^
15–19 years	16	5.3	2.7	4.0	–	0.228 ^*^
20–24 years	69	22.8	17.9	19.1	–	
25–29 years	86	28.5	41.2	30.2	–	
30–34 years	89	29.5	23.5	29.3	–	
≥35 years	42	13.9	14.7	17.4	–	
Sector of residence						<0.001^**^
Urban	66	21.9	–	70.4	–	
Rural	236	78.1	–	29.5	–	
Ethnicity						<0.001^**^
Sinhalese	199	65.9	–	72.2	–	
Sri Lankan Tamils	61	20.2	–	10.3	–	
Sri Lankan Moors	42	13.9	–	12.5	–	
Indian Tamil	0	0	–	4.5	–	
Others	0	0	–	0.5	–	
Education						
Primary education	5	1.7	–	–	–	
Secondary education	258	85.4	–	–	–	
Tertiary education	39	12.9	–	–	–	
Employment						
Full-time paid work	52	17.2	–	–	–	
Part-time paid work	4	1.3	–	–	–	
Casual paid work	2	0.7	–	–	–	
Self-employed	4	1.3	–	–	–	
Unemployed	240	79.5	–	–	–	
Household income						
<LKR 100,000	6	2.0	–	–	–	
LKR 100,000–500,000	184	60.9	–	–	–	
LKR 501,000–1,000,000	79	26.2	–	–	–	
>LKR 1,000,000	22	7.3	–	–	–	
Do not know/remember	11	3.6	–	–	–	
***Pregnancy***						
Pre-pregnancy BMI						<0.001^†††^
< 18.5 kg/m^2^	36	11.9	–	–	15.5^c†††^	
18.5-22.9 kg/m^2^	100	33.1	–	–	54.6 ^c†††^	
23-27.49 kg/m^2^	108	35.8	–	–	^##^29.9 ^c†††^	
≥27.5 kg/m^2^	39	12.9	–	–		
Not recorded	19	6.3	–	–		
Parity						<0.001^**†††**^
Primipara	129	42.7	33.4		32.3^c**†††**^	
Multiparous	173	57.3	66.6		67.7^c**†††**^	
Antenatal clinic						<0.001^**†††**^
Visited	301	99.7	–	–	95.4^c**†††**^	
Not visited	1	0.3	–	–	4.6^c**†††**^	
Antenatal Morbidities						<0.001^**†††**^
Anaemia	21	7.0	–	–	26.9^c**†††**^	
Gestational Diabetes Mellitus	21	7.0	–	–	5.3^c**†††**^	
Pregnancy-induced	14	4.6	–	–	2.8^c**†††**^	
Heart Disease	1	0.3	–	–	0.5^c**†††**^	
Urinary Tract Infection	1	0.3	–	–	–	
Epilepsies	1	0.3	–	–	–	
Chronic Diabetes Mellitus	7	2.3	–	–	1.1^c**†††**^	
Asthma	4	1.3	–	–	–	
Chronic Hypertension	2	0.7	–	–	0.3^c**†††**^	
***Childbirth***						
Mode of birth						0.004^*^
Unassisted vaginal birth	192	63.6	58.7	–	–	
Vaginal birth assisted with forceps	1	0.3	0.8	–	–	
Vaginal birth assisted with vacuum	5	1.7	1.2	–	–	
Vaginal birth assisted with forceps and vacuum	5	1.7	0	–	–	
Elective caesarean section	76	25.2	23.6	–	^#^40.5^c**†**^	
Emergency caesarean section	23	7.6	15.7	–		
Medication during labor						
Administered	275	91.1	–	–	–	
No data available	27	8.9	–	–	–	
Gestational age at birth						
< 37 weeks	20	6.6	9.0	–	–	0.145^*^
≥ 37 weeks	282	93.4	91.4	–	–	
Gestation						0.39^*^
Singleton	300	99.3	98.8	–	–	
Multiple	2	0.7	1.2	–	–	
Baby’s weight at birth						0.004^*^
< 2500 grams	28	9.3	15.3	–	12.1^c**††**^	<0.001^**††**^
≥2500 grams	274	90.7	84.7	–	88.9^c**††**^	
Maternal Morbidities or complications reported (labor and childbirth)						
Haemorrhage	10	3.3	2.0	–	0.6^c**†**^	
Poor progression of labor	8	2.6	–	–	–	
Infection	1	0.3	–	–	–	
Cardio-respiratory discomfort	2	0.7	–	–	–	
Foetal/Neonatal complications reported						
No	275	91.1	–	–	–	
Yes	27	8.9	–	–	–	

^a^Women who gave birth to a live baby in selected hospitals between September and December 2021.

^b^Live births, registered in Sri Lanka, by the Department of Registrar General, Sri Lanka in 2015 [18]. The number of live births were considered as the total population of birthing women because data excluding stillbirths is not available.

^c^† Births in government health facilities in 2019, reported by Family Health Bureau, Sri Lanka [11].

^c^†† Births registered by Public Health Midwives in 2019, reported by Family Health Bureau, Sri Lanka [11].

^c^††† Pregnant mothers registered by Public Health Midwives in 2019, reported by Family Health Bureau, Sri Lanka [11].

^*^Compared to the population of women who gave birth to a live baby in selected hospitals between September and December 2021.

^**^Compared to the number of live births, registered in Sri Lanka, by the Department of Registrar General, Sri Lanka in 2015 [18].

^††^Compared to the number of live births registered by Public Health Midwives in 2019, as reported by the Family Health Bureau, Sri Lanka [11].

^†††^Compared to the number of pregnant mothers registered by Public Health Midwives in 2019 as reported by the Family Health Bureau, Sri Lanka [11].

^#^Number/percentage of caesarean sections that occurred (both elective and emergency) in Sri Lankan health facilities.

^##^Pregnant women with a Body Mass Index (BMI) higher than 23 kg/m^2^.

The sample was under-represented by 25 to 29-year-old women, and over-represented women aged under 25 years when compared to all women who gave birth in the selected hospitals but was representative of the national population [18] in terms of age. Women living in rural areas and Sri Lankan Tamil women were over-represented, and Sinhalese women were under-represented, compared to the national distribution of women who give birth [18].

Women with a BMI greater than 23 kg/m^2^, who had gestational diabetes mellitus (GDM), anemia, and pregnancy complications were over-represented in our sample, and more women attended antenatal clinic visits compared to the national distribution of pregnant women in Sri Lanka [11]. The proportion of women who gave birth to a single baby after 37 weeks of gestation was similar in our selected hospitals. Multiparous women, who had a vaginal birth, and had a baby that weighed over or equal to 2500 grams were over-represented in our sample compared to women who gave birth in the selected hospitals and the national distribution [11] (Table 1).

Eleven women did not know or remember the time when their baby was fed colostrum for the very first time after birth and were excluded, leaving data from 291 women that were used for measuring associations with early initiation of breastfeeding. Early initiation of breastfeeding was reported by 64.5% of women. Assumptions for logistic regression were met by the data with no evidence of multi-collinearity between variables, and observations independent of each other.

Associations with early initiation of breastfeeding are presented in Table 2. In univariate regression, only BMI under 18 kg/m^2^ significantly increased the odds of early initiation of breastfeeding. Women aged over 35 years, who were of Sri Lankan Tamil ethnicity, had an elective or emergency caesarean section, were administered spinal anaesthesia and antibiotics, and whose babies had respiratory distress after birth had decreased odds of early initiation of breastfeeding (Table 2). After adding all variables to the regression model simultaneously, only BMI under 18 kg/m^2^ (OR: 6.17, 95% CI: 1.42–26.81) and elective caesarean section (OR: 0.08, 95% CI: 0.01–0.43) remained significant.

**Table 2:** Association between early breastfeeding initiation, and demographic and clinical characteristics; results of all three modelling approaches presented.

Participants Characteristics	Breastfeeding initiation								
	Early breastfeeding initiation % (n = 195)	No Early breastfeeding initiation % (n = 96)	Bivariate model		Multivariable model			Final model	
			OR	(95% CI)	aOR	(95% CI)	aOR		(95% CI)
***Socio-demographics***									
Age									
25–29 years	32.3	21.9	1	ref	1	ref			
20–24 years	21.5	24.0	0.61	(0.30-1.24)	0.41	(0.15-1.13)	–		–
30–34 years	30.3	27.1	0.76	(0.39-1.49)	0.92	(0.38-2.24)	–		–
≥ 35 years	10.8	21.9	0.33	(0.15-0.73)^**^	0.48	(0.16-1.41)	–		–
15–19 years	5.1	5.2	0.67	(0.20-2.17)	0.52	(0.10-2.71)	–		–
Sector of residence									
Rural	80.5	76.0	1	ref	1	ref	–		–
Urban	19.5	24.0	0.77	(0.43-1.38)	0.57	(0.24-1.36)	–		–
Ethnicity									
Sinhalese	69.2	56.3	1	ref	1	ref	–		–
Sri Lankan Tamils	17.0	28.1	0.49	(0.27-0.89)^*^	0.82	(0.33-2.05)	–		–
Sri Lankan Moors	13.8	15.6	0.72	(0.36-1.46)	0.82	(0.29-2.36)	–		–
Education									
Secondary education	84.1	87.5	1	ref	1	ref	–		–
Tertiary education	14.4	10.4	1.4	(0.67-3.09)	2.08	(0.55-7.94)	–		–
Primary education	1.5	2.1	0.77	(0.13-4.69)	2.09	(0.25-16.94)	–		–
Employment									
Unemployed	80.0	77.1	1	ref	1	ref	–		–
Full-time paid work	17.4	17.7	0.95	(0.50-1.81)	0.29	(0.08-1.06)	–		–
Part-time paid work	1.0	2.1	0.47	(0.07-3.43)	0.19	(0.02-1.80)	–		–
Casual paid work	0.6	1.0	0.47	(0.03-7.69)	0.56	(0.02-14.26)	–		–
Self-employed	1.0	2.1	0.47	(0.07-3.43)	0.11	(0.01-2.37)	–		–
Household income									
LKR 100,000–500,000	58.5	68.8	1	ref	1	ref			
LKR 501,000–1,000,000	28.7	21.9	1.54	(0.86-2.77)	2.62	(1.05-6.59)^*^	–		–
>LKR 1,000,000	8.7	3.1	3.28	(0.93-11.61)	6.67	(1.14-39.19)^*^	–		–
<LKR 100,000	1.5	3.1	0.58	(0.11-2.95)	0.52	(0.08-3.47)	–		–
Unknown	2.6	3.1	0.97	(0.22-4.17)	1.02	(0.12-8.83)	–		–
***Pregnancy***									
Pre-pregnancy BMI									
18.5-22.9 kg/m^2^	32.3	33.3	1	ref	1	ref	1		ref
< 18.5 kg/m^2^	14.9	5.2	2.95	(1.04-8.34)^*^	6.17	(1.42-26.81)^*^	4.17		(1.23-14.05)^*^
23-27.49 kg/m^2^	35.9	36.5	1.02	(0.56-1.83)	0.96	(0.44-2.07)	1.08		(0.56-2.08)
≥27.5 kg/m^2^	12.8	14.6	0.91	(0.42-1.98)	0.72	(0.25-2.07)	1.15		(0.48-2.75)
Don’t know (Not recorded)	4.1	10.4	0.41	(0.15-1.13)	0.23	(0.06-0.87)^*^	0.31		(0.10-0.92)^*^
Parity									
Multiparous	56.9	60.4	1	ref	1	ref	–		–
Primipara	43.1	39.6	1.15	(0.70-1.90)	1.21	(0.54-2.70)	–		–
Antenatal Morbidities/complications^**†**^									
Anaemia	7.2	5.2	1.41	(0.49-4.03)	0.75	(0.16-3.45)	–		–
Gestational Diabetes Mellitus	6.2	8.3	0.72	(0.29-1.83)	1.09	(0.32-3.69)	–		–
Pregnancy-induced	3.6	5.2	0.68	(0.21-2.19)	0.84	(0.17-3.97)	–		–
Chronic Diabetes Mellitus	2.1	3.1	0.65	(0.14-2.96)	0.97	(0.09-10.32)	–		–
Asthma	1.0	2.1	0.49	(0.07-3.51)	1.99	(0.17-23.75)	–		–
Chronic Hypertension	0.5	1.0	0.49	(0.03-7.91)	0.61	(0.00-156.423)	–		–
***Childbirth***									
Mode of birth									
Unassisted vaginal birth	73.8	41.7	1	ref	1	ref	1		ref
Elective caesarean section	17.4	43.8	0.23	(0.13-0.40)^***^	0.08	(0.01-0.43)^**^	0.27		(0.14-0.51)^***^
Emergency caesarean section	5.6	9.4	0.34	(0.13-0.88)^*^	0.30	(0.06-1.57)	0.52		(0.18-1.56)
Vaginal birth assisted with forceps and vacuum	1.5	2.1	0.42	(0.07-2.58)	0.36	(0.03-4.38)	0.60		(0.08-4.71)
Vaginal birth assisted with vacuum	1.5	2.1	0.42	(0.07-2.58)	2.01	(0.21-19.81)	0.83		(0.12-5.87)
Vaginal birth assisted with forceps	0	1.0	#	#	#	
Medication administered during labor^**†**^									
Intravenous fluid	82.1	84.4	0.85	(0.44-1.64)	0.61	(0.19-2.0)	–		–
Labor inducers/augmenting agents	71.3	32.5	1.49	(0.89-2.50)	0.56	(0.18-1.74)	–		–
Analgesics	21.0	13.5	1.70	(0.86-3.35)	0.67	(0.20-2.12)	–		–
Antiemetics	19.5	17.7	1.13	(0.60-2.12)	1.46	(0.47-4.49)	–		–
Spinal anaesthesia	16.9	42.7	0.27	(0.16-0.47)^***^	1.71	(0.37-7.88)	–		–
Antibiotics	9.7	30.2	0.25	(0.13-0.48)^***^	0.38	(0.13-1.16)	0.40		(0.19-0.87)^*^
Gestational age at birth									
≥ 37 weeks	94.4	92.7	1	ref	1	ref	–		–
< 37 weeks	5.6	7.3	0.76	(0.29-2.03)	0.90	(0.23-3.59)	–		–
Baby’s weight at birth									
≥ 2500 grams	92.3	87.5	1	ref	1	ref	–		–
< 2500 grams	7.7	12.5	0.58	(0.26-1.30)	0.51	(0.16-1.59)	–		–
Maternal Morbidities/discomforts^**†**^ (labor and childbirth)									
Haemorrhage	2.6	5.2	0.48	(0.14-1.70)	0.26	(0.06-1.24)	–		–
Poor progression of labor	2.1	4.2	0.48	(0.10-2.45)	0.25	(0.04-1.74)	–		–
Foetal/neonatal morbidities/complications^**†**^									
Not cried at birth	3.6	6.3	0.82	(0.19-83.49)	1.50	(0.21-10.56)	–		–
Jaundice	2.1	4.2	0.48	(0.12-1.969	0.51	(0.06-4.34)	–		–
Respiratory distress	1.0	6.3	0.16	(0.03-0.79)^**^	0.05	(0.00-0.59)^*^	0.08		(0.01-0.57)^*^
Increased/decreased heartbeat	0.5	1.0	0.49	(0.03-7.91)	1.03	(0.03-33.54)	–		–
Sepsis	0.5	2.1	0.24	(0.02-2.71)	0.08	(0.00-1.52)	–		–
Growth/Birth defect	0.5	2.1	0.24	(0.02-2.71)	0.51	(0.01-30.20)	–		–

OR - Odds Ratio; aOR - adjusted Odds Ratio; CI - Confidence Interval Significance at *p < 0.05; **p < 0.01; ***p < 0.001.

^†^Variables in which ‘no’ is the referent.

^#^Not calculable.

Following backward step-wise elimination of the least significant independent variables listed in Table 2, variables that had the most significant association with early initiation of breastfeeding were identified in the final model, which was highly parsimonious and explained 17.9–24.9% of the variance in early initiation of breastfeeding (Nagelkerke’s pseudo-R^2^ at 0.249 and Cox and Snell pseudo R^2^ at 0.179). Women with a BMI under 18.5 kg/m^2^ had higher odds of early initiation of breastfeeding (aOR: 4.17, 95% CI: 1.23–14.05) than women with a BMI of 18.5 to 22.9 kg/m^2^. Women who had an elective caesarean section had lower odds of early initiation of breastfeeding (aOR: 0.27; 95% CI: 0.14–0.51) than women who had a vaginal birth. Women who were administered antibiotics (aOR: 0.4; 95% CI: 0.19–0.87) or had a baby with respiratory distress (aOR: 0.08, 95% CI: 0.01–0.57) had lower odds of early initiation of breastfeeding than those who were not administered medication or who had babies with no complications after birth, respectively.

## Discussion

This is the first known study conducted in Sri Lankan government hospitals to assess the association between socio-demographic and clinical characteristics related to pregnancy and childbirth and early initiation of breastfeeding. Findings revealed that 64.5% of women started early breastfeeding, which was higher than the prevalence reported in South Asian countries [3,19] and globally [19], but lower than the reported national prevalence in Sri Lanka (90.3%) [12]. Our estimates may be more reliable than alternative data collections because women were approached close to childbirth. The reported national prevalence of early initiation of breastfeeding in Sri Lanka and other countries is derived from women’s self-reports from a few months to years after giving birth [3,12]. Maternal short-term recall of breastfeeding initiation is more reliable due to recall bias towards over-estimating breastfeeding practices when surveyed days or months after giving birth [20]. In our study, data were collected by an independent researcher, however, the national survey is conducted by the government. Therefore, women may report to the government what they think the government wants to hear. Elective caesarean section, administration of antibiotics, and respiratory distress in neonates after birth were significant negative predictors of early initiation of breastfeeding. Maternal BMI under 18 kg/m^2^ was a significant positive predictor of early initiation of breastfeeding. Together, these variables explained 17.9–24.9% of the variance in initiation of breastfeeding. However, the total variance explained in our model cannot be contextualised because similar research in the region [3,9] fails to report the amount of variation explained by the predictors examined. Findings that women who gave birth by elective caesarean section were less likely to initiate early breastfeeding are consistent with studies conducted in Canada [7] and Bangladesh [21]. In contrast, Zanardo et al [22] reported that early initiation of breastfeeding is less likely after an emergency caesarean section. Regardless of whether it was planned or unplanned, caesarean section is considered a major barrier to early initiation of breastfeeding in Sri Lanka [9]. This may be due to delayed mother-baby skin-to-skin contact because of post-operative care or increased maternal stress, especially in emergency caesarean sections [21,23]. Elective caesarean section may reduce the likelihood of early initiation of breastfeeding as an absence of labor means there are fewer hormonal drivers responsible for lactogenesis [7,22]. Women who undergo elective caesarean section are also more likely to have neonates who are between 34 and less than 37 weeks gestation, with poor sucking skills, lower birth weight, and low level of alertness which can affect early initiation of breastfeeding [7].

We found that women who had babies with respiratory distress were less likely to initiate early breastfeeding, regardless of gestational age. Similar results were observed in babies born in Bangladesh [21] who had asphyxia. In these scenarios, mothers and babies may be separated due to medical interventions, and breastfeeding may not be recommended until recovery [21]. We also found that women who were administered antibiotics were less likely to initiate breastfeeding early. Antibiotic prophylaxis for women undergoing caesarean section is a global recommendation [24]. Antibiotics are administered as prophylaxis before/after caesarean section to minimize the risks of post-operative infections, such as endometritis, surgical site infections, and urinary tract infections [24,25]. In this study, most participants who gave birth by caesarean section had been given antibiotics as is common practice in Sri Lanka [26]. However, antibiotics are compatible with lactogenesis [27]. Therefore, we assume that delayed initiation of breastfeeding in women who were administered antibiotics in our study might be an additional effect associated with the circumstances related to caesarean section.

In our study, we have strengthened the theory that caesarean section is associated with delayed breastfeeding initiation, particularly in Sri Lanka, due to our wide sampling frame, high response rate, and results that are mostly consistent with others internationally. Women who give birth by caesarean section in Sri Lanka should receive targeted support to initiate breastfeeding early, as elective caesarean section is a barrier in multiple ways. For example, mother-baby skin-to-skin contact is often delayed following elective caesarean section because of post-operative care or increased maternal stress [21,23]. Elective caesarean section may reduce the likelihood of early initiation of breastfeeding as an absence of labour means there are fewer hormonal drivers responsible for lactogenesis [7,22]. Also, women who undergo elective caesarean section are more likely to have neonates who are between 34 and less than 37 weeks gestation, with poor sucking skills, lower birth weight and low level of alertness which can affect early initiation of breastfeeding [7]. Furthermore, as the rate of caesarean section is increasing in Sri Lanka [28], the risk of delayed breastfeeding initiation is likely to persist unless mitigating support programs are designed, funded, and implemented.

In our study, women with a BMI under 18 kg/m^2^ were more likely to initiate early breastfeeding compared to women with a BMI of 18.5 to 22.9 kg/m^2^ – normal weight. In Italy, Giovannini et al [29] also found that underweight women were more likely to put babies on their breasts as early as possible than normal-weight women. Underweight women were more likely to be primiparous and have a vaginal birth, and less likely to birth underweight babies and have gestational diabetes mellitus and gestational hypertension [29]. Significant associations between early initiation of breastfeeding and these factors have been previously established [9,30,31]. However, Tao et al [32] reported no significant variations in the early initiation of breastfeeding across pre-pregnancy BMI in China. There is also contrasting evidence that a BMI under 18 kg/m^2^ may be negatively associated with early initiation of breastfeeding [33]. In other studies [8,34] that reported negative associations between pre-pregnancy underweight and early initiation of breastfeeding, BMI was calculated using women’s self-reports of weight and height. Women usually overstate their height and under report their weight, which may result in the incorrect calculation of BMI [31], and introduce mis-classification bias in assessing associations between women’s BMI and early breastfeeding. We obtained BMI data from women’s medical records, which are generally determined by health staff directly measuring women’s weight and height. The different methods for measuring BMI may explain the variation in results across studies.

### Strengths and limitations

In our study, Sri Lankan women who gave birth in selected government hospitals were approached close to childbirth. Therefore, we were able to minimize over-reporting of early breastfeeding practices associated with later recall [20]. Also, this study included information on early initiation of breastfeeding that occurred after the recent childbirth of participating women, which improved the precision of data. Our data on women’s characteristics and early breastfeeding practices is likely to be more reliable than inconsistently documented Sri Lankan patient admission data, since maternal self-report data about newborn care is more reliable than clinical records [35]. We provided opportunities for all eligible women to participate in this study, and therefore, this study represented women with various socio-demographic backgrounds who gave birth in the selected hospitals. We collected data using an interviewer-administered survey, which minimised missing information related to carelessness or potential misinterpretation by the respondent. Our sample of 291 women exceeded the minimum sample size required to confidently assess the associations in our study. Also, results and representativeness reported in this study are valid as no participants dropped out of the study at any stages, and data are consistent.

However, this study was conducted in only four hospitals which affected the generalisability of the study to all of Sri Lanka, and population data was not available for all relevant characteristics to comprehensively assess sample representativeness. Further, findings reported with wide confidence intervals across the variables presented in this study may indicate the inadequacy of the samples included in the logistic regression analysis. This may impact the credibility of the data reported, and caution should be applied when translating our findings to the broader context. Also, the significant predictors of early initiation of breastfeeding, especially pre-pregnancy BMI of women and administration of antibiotics, can only be tentatively considered and may not apply to the wider population, particularly given their inconsistency with existing evidence [27,33]. Therefore, further research may be needed across the country for reviewing and revising policies on breastfeeding support care that target all at-risk women in Sri Lanka.

## Conclusion

The findings reported in this study emphasise that health facilities and community health service units need to recognise the unique traits and risk factors of women who seek prenatal and childbirth care, to provide appropriate care that minimises obstacles to breastfeeding and the well-being of mothers and newborns. The Sri Lankan health system can review the implementation of Baby-Friendly Hospital Initiative practices, including facilitating mother-baby skin-to-skin contact, educating breastfeeding techniques and responsive feeding, facilitating rooming-in in hospitals to support women and practicing early initiation of breastfeeding. Women should be adequately informed of the possible risks associated with medications administered during labour and childbirth on early postpartum recovery and supported to make informed decisions about the use of medication and manage the impact on early initiation of breastfeeding [36].

Healthcare professional organisations and regulatory bodies in Sri Lanka can offer continuous education and training for doctors, nurses, and midwives on the significance and best practice of facilitating mother-baby skin-to-skin contact following birth, particularly for caesarean births, while also performing other postpartum care [23]. These training programs need to be planned, designed, and implemented very soon in Sri Lanka, as the trend of delayed breastfeeding initiation in Sri Lanka may persist due to the increasing rates of caesarean section [11]. Furthermore, there is a need for implementing research in a broader context in the future to validate the findings of this study and to evaluate how well breastfeeding support practices, such as the Baby-Friendly Hospital Initiative, are implemented in Sri Lankan health facilities to minimise disparities in early initiation of breastfeeding associated with significant predictors [37].

## Supporting information

S1 Data Data used in this study. (XLSX)
